# The application value of serum Th1/Th2 cytokines combined with tumour markers in the diagnosis of HR-HPV-positive cervical cancer

**DOI:** 10.5937/jomb0-59352

**Published:** 2026-03-17

**Authors:** Xia Cao, Xuemei Zhang, Jingpo Zhang, Xiaodang Zhong, Shanshan Hu

**Affiliations:** 1 Danyang Maternal and Child Health Care Hospital, Department of Gynaecology, China; 2 Ezhou Central Hospital, Department of Oncology, China; 3 First Hospital of Hebei Medical University, Obstetrics and Gynaecology, China; 4 Huazhong University of Science and Technology, Hubei Cancer Hospital, Department of Gynaecological Oncology, Tongji Medical College, China

**Keywords:** serum Th1/Th2 cytokines, tumour markers, diagnosis, HR-HPV-positive, cervical cancer, Th1/Th2 citokini u serumu, tumorski markeri, dijagnoza, HR-HPV pozitivno, karcinom grlica materice

## Abstract

**Background:**

To determine the efficacy of tumour markers in conjunction with serum helper T-cell 1 (Th1) and helper T-cell 2 (Th2) cytokines for the detection of HR-HPV-positive cervical cancer.

**Methods:**

49 patients with HR-HPV-positive benign cervical diseases of the same age group who were admitted to the hospital between January 2020 and June 2024 were chosen as the control group, and 49 patients with HR-HPV-positive cervical cancer were chosen as the study group. Serum Th1/Th2 cytokine levels of patients with various clinicopathological characteristics in the study group were compared with the basic data, tumour markers, and serum Th1/Th2 cytokines [interleukin (IL)-10, IL-6, interferon-g (IFN-g), and tumour necrosis factor-a (TNF-<span style="color: rgb(32, 33, 34); font-family: sans-serif; font-size: 16px; background-color: rgb(248, 249, 250);">α</span>)] and tumour markers between the two groups. The association between tumour markers and serum Th1/Th2 cytokine levels in patients with HR-HPV-positive cervical cancer was examined using Pearson correlation analysis.

**Results:**

International Federation of Obstetrics and Gynecology (FIGO) stages III-IV patients in the study group had greater serum levels of IL-10, IL-6, IFN-g, and TNF-<span style="color: rgb(32, 33, 34); font-family: sans-serif; font-size: 16px; background-color: rgb(248, 249, 250);">α</span> than the control group (P&lt; 0.05), and the levels of these substances in the patients with a myometrial invasion depth 1/2 were all higher than in the patients with a myometrial invasion depth &lt;1/2 (P&lt; 0.05). SCC-Ag, CA199, and there was a significant correlation (P&lt; 0.05) between CYFRA21-1 levels and blood levels of IL-10, IL-6, IFN-g, and TNF-<span style="color: rgb(32, 33, 34); font-family: sans-serif; font-size: 16px; background-color: rgb(248, 249, 250);">α</span> in patients with HR-HPV-positive cervical cancer. The AUCs of IL-10, IL-6, IFN-g and TNF-<span style="color: rgb(32, 33, 34); font-family: sans-serif; font-size: 16px; background-color: rgb(248, 249, 250);">α</span> for the single diagnosis of HR-HPV-positive cervical cancer were 0.809, 0.773, 0.801 and 0.794, respectively, and the AUCs of SCC-Ag, CA199 and CYFRA21-1 for the single diagnosis of HR-HPV-positive cervical cancer were 0.831 and 0, respectively. 728, 0.789. The AUC of the combined diagnosis of HR-HPV-positive cervical cancer by serum IL-10, IL-6, IFN-g, TNF-<span style="color: rgb(32, 33, 34); font-family: sans-serif; font-size: 16px; background-color: rgb(248, 249, 250);">α</span>, SCC-Ag, CA199, and CYFRA21-1 was 0.927. The AUC of the combined diagnosis was greater than that of the individual diagnosis of each index (Z = 2 .1 16, 2.690, 2.341, 2.565, 1.957, 3.351, 2.631, P= 0.034, 0.007, 0.019, 0.010, 0.039, 0.001, 0.009).

**Conclusions:**

The combined detection of serum Th1/Th2 cytokine imbalance (elevated IL-6 and IL-10) and tumour markers (SCC, CA125) can significantly improve the diagnostic accuracy of HR-HPV-positive cervical cancer and provide a high-value serological auxiliary basis for the early identification of cervical cancer progression.

## Abstract (sr)

**Uvod:**

Cilj je bio da se ispita efikasnost tumorskih markerau kombinaciji sa serumskim citokinima pomoćničkih Tćelija tipa 1 (Th1) i tipa 2 (Th2) u detekciji karcinoma grlićamaterice pozitivnog na visokorizični humani papiloma virus(HR-HPV).

**Metode:**

Kontrolnu grupu činilo je 49 pacijentkinja sa HRHPV pozitivnim benignim oboljenjima grlića materice, istogstarosnog doba, koje su hospitalizovane u periodu od januara 2020. do juna 2024. godine. Ispitivanu grupu činiloje 49 pacijentkinja sa HR-HPV pozitivnim karcinomomgrlića materice. Upoređeni su nivoi Th1/Th2 citokina userumu pacijentkinja različitih kliničko-patoloških karakteristika iz ispitivane grupe sa osnovnim podacima, tumorskimmarkerima i serumskim Th1/Th2 citokinima [interleukin(IL)-10, IL-6, interferon-g (IFN-g) i faktor nekroze tumoraalfa (TNF-<span style="color: rgb(32, 33, 34); font-family: sans-serif; font-size: 16px; background-color: rgb(248, 249, 250);">α</span>)] između dve grupe. Veza između tumorskihmarkera i nivoa Th1/Th2 citokina u serumu kod pacijentkinja sa HR-HPV pozitivnim karcinomom grlića matericeispitana je Pirsonovom korelacionom analizom.

**Rezultati:**

Pacijentkinje u FIGO stadijumima III-IV u ispitivanoj grupi imale su značajno vise serumske nivoe IL-10,IL-6, IFN-g i TNF-<span style="color: rgb(32, 33, 34); font-family: sans-serif; font-size: 16px; background-color: rgb(248, 249, 250);">α</span> u poređenju sa kontrolnom grupom (P&lt; 0,05). Takođe, ovi nivoi su bili viši kod pacijentkinja sadubinom invazije u miometrijum 1/2 u poređenju saonima kod kojih je invazija bila &lt;1/2 (P&lt; 0,05). Utvrđenaje značajna korelacija (P&lt; 0,05) između nivoa SCC-Ag,CA199 i CYFRA21-1 i nivoa IL-10, IL-6, IFN-g i TNF-<span style="color: rgb(32, 33, 34); font-family: sans-serif; font-size: 16px; background-color: rgb(248, 249, 250);">α</span> userumu kod pacijentkinja sa HR-HPV pozitivnim karcinomom grlica materice. AUC (area under the curve) zapojedinačne dijagnostičke pokazatelje iznosio je 0,809 zaIL-10, 0,773 za IL-6, 0,801 za IFN-g i 0,794 za TNF-<span style="color: rgb(32, 33, 34); font-family: sans-serif; font-size: 16px; background-color: rgb(248, 249, 250);">α</span>,dok su AUC vrednosti za SCC-Ag, CA199 i CYFRA21-1bile 0,831, 0,728 i 0,789. Kombinovana dijagnostičkaAUC vrednost za IL-10, IL-6, IFN-g, TNF-<span style="color: rgb(32, 33, 34); font-family: sans-serif; font-size: 16px; background-color: rgb(248, 249, 250);">α</span>, SCC-Ag,CA199 i CYFRA21-1 iznosila je 0,927, sto je bilo značajnovise u poređenju sa pojedinačnim pokazateljima(Z = 2,116; 2,690; 2,341; 2,565; 1,957; 3,351; 2,631;P= 0,034; 0,007; 0,019; 0,010; 0,039; 0,001; 0,009).

**Zaključak:**

Kombinovano određivanje neravnote eTh1/Th2 citokina u serumu (povecani IL-6 i IL-10) i tumorskih markera (SCC, CA125) može značajno poboljšatitačnost dijagnoze HR-HPV pozitivnog karcinoma grlicamaterice i predstavlja značajan seroloski alat u ranom otkrivanju progresije bolesti.

## Introduction

The age-standardised incidence rate of cervical cancer among Chinese women increased by 3.7% annually, and the annual growth rate of the age-standardised mortality rate was approximately 3.6% [1][2][3]. The prevention and treatment situation of cervical cancer is severe [4]. At present, serum tumour markers are widely used in the diagnosis and treatment of cervical cancer. However, false positives still exist in their diagnosis. The diagnostic efficacy of these methods is not high [5][6], and new indicators for the early diagnosis of cervical cancer caused by HPV infection still need to be identified to compensate for the deficiencies of the existing indicators in diagnosis. Cervical cancer generally has disorders of helper T-cell 1 (Th1)/helper T-cell 2 (Th2)-related cytokines, which may promote the occurrence and development of cervical cancer [7]. Moreover, studies have shown that in clinical practice, early diagnosis and treatment plans can be formulated by detecting the levels of Th1/Th2-related cytokines in patients with cervical cancer to improve the prognosis of patients [8]. However, the role of Th1/Th2 cytokines in the diagnosis of cervical cancer remains unclear.

Therefore, our study sought to determine whether serum Th1/Th2 cytokines in conjunction with tumour markers may be used to diagnose HR-HPV-positive cervical cancer.

## Materials and methods

### General information

Forty-nine patients with HR-HPV-positive cervical cancer admitted to our hospital from January 2020 to June 2024 were selected as the study group, and all patients met the diagnostic criteria for cervical cancer.

Forty-nine patients with HR-HPV-positive benign cervical diseases of the same age group were admitted to our hospital, and all were diagnosed with benign cervical diseases by histopathological examination.

Inclusion criteria: (1) Aged 18-75 years; (2) HR-HPV positive; (3) Had a history of sexual activity but nearly 3d asexual lifestyle; (4) Had not yet received relevant treatment.

Exclusion criteria: (1) Had a history of vaginal medication in the past 3 weeks; (2) Had a history of severe infection, trauma or major surgery in the past three months; (3) Had a mental illness or communication disorder; (4) Had blood system diseases; (5) Had severe organ dysfunction; (6) Suffered from other malignant tumours; (7) Had a history of surgeries such as conisation of the cervix and hysterectomy; (8) Pregnant or lactating; (9) Had severe endocrine, immune or other system diseases.

### Detection of Th1/Th2 cytokines and tumour markers

After centrifugation (Rotational speed: 3500 r/min, 10 min), the serum was collected. It was a Spectra Max190 microplate reader (MD Company, USA) that was used for detection. The electrochemiluminescence method (kit manufacturer: Shanghai Lanji Biotechnology Co., Ltd.) was used to measure the levels of serum SCC-Ag, CA199, and CYFRA21-1, and the detection instrument used was a DX1800 fully automatic electrochemiluminescence immuno-analyzer (Beckman Coulter, USA).

### Data collection

Data such as age, body mass index, history of miscarriage, birth frequency, age of menarche, history of alcohol consumption, family history of cervical cancer, underlying diseases (Diabetes, hypertension, hyperlipidemia), menopausal status, and vaginal microecological imbalance were collected for all patients.

Vaginal microecological results: In a normal microecological environment, the pH ranged from 3.8 to 4.5, and the concentration of hydrogen peroxide was negative. β-glucuronidase negative; negative for leukocyte esterase; negative for sialidase activity; negative for β-galactosidase; negative for Candida and Trichomonas. These abnormalities indicate an imbalance in the vaginal microecology.

### Statistical processing

Software called SPSS 27.0 was used to process the data. For group comparisons, the 2 test was employed, and count statistics are presented as percentages and the number of cases. The association between serum Th1/Th2 cytokine levels and tumour markers in patients with HR-HPV-positive cervical cancer was examined using Pearson correlation analysis. When diagnosing HR-HPV-positive cervical cancer, the value of serum Th1/Th2 cytokines and tumour markers was analysed using the ROC curve. The DeLong test was utilised to compare the area under the curve (AUC). P values less than 0.05 were regarded as statistically significant.

## Results

### Comparison of the general data of the two groups

Age, body mass index, parity, smoking, drinking, family history of cervical cancer, underlying conditions, menopause, and history of miscarriage did not differ statistically significantly (P>0.05). The variations in menarche rates were statistically significant (Age ≤13 years) and vaginal microecological imbalance between the two groups (P<0.05). See Table 1 and Table 2.

**Table 1:** Comparison of general data [x̄±s, n(%)].

Group	n	Age (years)	Body mass<br>index (kg/m^2^)	Have a history <br>of miscarriage	Production<br>frequency (times)	Age of menarche<br>≤13 years	Have a history<br>of smoking
Research group	49	52.01±7.69	23.35±1.74	10 (20.41)	3.16±0.82	21 (42.86)	12 (24.49)
Control group	49	49.85±8.12	23.01±1.68	5 (10.20)	2.89±0.75	36 (73.47)	8 (16.33)
t/x^2^		1.352	0.984	1.968	1.701	9.435	1.005
P		0.180	0.328	0.161	0.092	0.002	0.316

**Table 2:** Comparison of patient case data of the two groups [x̄±s or n(%)].

Group	n	Have a<br>history of<br>drinking<br>alcohol	Have a<br>family history<br>of cervical<br>cancer	Have<br>diabetes	Have<br>high blood<br>pressure	Have<br>hyperlipidemia	Menopause	Vaginal<br>microecological<br>imbalance
Research <br>group	49	9 (18.37)	6 (12 .24)	8 (16.33)	11 (22.45)	6 (12.24)	31 (63.27)	33 (67.35)
Control<br>group	49	5 (10.20)	2 (4.08)	5 (10.20)	9 (18.37)	8 (16.33)	25 (51.02)	20 (40.82)
t/x^2^		1.333	1.225	0.798	0.251	0.333	1.5	6.944
P		0.248	0.268	0.372	0.616	0.564	0.221	0.008

### Comparison of Th1/Th2 cytokine and tumour marker levels between the two groups

The levels of serum IL-10, IL-6, IFN-γ, TNF-α, SCC-Ag, CA199 and CYFRA21-1 in the study group were all greater than those in the control group (P<0.05). See Table 3.

**Table 3:** Comparison of Th1/Th2 cytokine and tumour marker levels in the two groups (x̄±s).

Group	n	IL-10<br>(pg/mL)	IL-6<br>(pg/mL)	IFN-γ<br>(pg/mL)	TNF-α<br>(pg/mL)	SCC-Ag<br>(ng/mL)	CA 199<br>(U/m L)	CYFRA21-1<br>(ng/mL)
Research <br>group	49	11.16±3.24	105.20±31.76	3.91±0.42	6.53±2.04	3.72±1.16	35.18±8.42	7.48±2.26
Control <br>group	49	6.92±2.18	61.24±18.43	3.24±0.38	3.17±0.95	1.83±0.54	20.76±6.70	2.18±0.61
t		7.600	8.380	8.281	10.452	10.340	9.381	15.849
P		<0.001	<0.001	<0.001	<0.001	<0.001	<0.001	<0.001

### Comparison of serum Th1/Th2 cytokine levels in patients with different clinicopathological characteristics in the study group

The levels of serum IL-10, IL-6, IFN-γ and TNF-α in patients with FIGO stage III and IV disease in the study group were greater than those in patients with stage and II disease (P<0.05). See Table 4 and Table 5.

**Table 4:** Comparison of serum Th1/Th2 cytokine levels in FIGO installment and degree of differentiation (x̄±s, pg/mL).

Clinicopathological characteristics	n	IL-10	IL-6	IFN-γ	TNF-α
FIGO Installment					
Stage I - II	30	9.75±2.85	94.37±25.14	3.68±0.35	5.71±1.84
Stage - IV	19	13.39±2.41	122.30±20.65	4.27±0.32	7.82±1.67
t		-4.615	-4.050	-5.939	-4.050
P		<0.001	<0.001	<0.001	<0.001
Degree of differentiation					
Moderate to low differentiation	33	11.36±3.11	106.84±30.25	3.96±0.39	6.62±1.95
High differentiation	16	10.75±2.79	101.82±27.51	3.81±0.35	6.34±1.71
t		0.665	0.560	1.304	0.490
P		0.509	0.578	0.199	0.627

**Table 5:** Comparison of serum Th1/Th2 cytokine levels in pathological type and depth of myometrial infiltration (x̄±s, pg/mL).

Clinicopathological characteristics	n	IL-10	IL-6	IFN-γ	TNF-α
Pathological type					
Squamous cell carcinoma	36	11.21±3.08	105.40±30.18	3.94±0.40	6.57±1.82
Adenocarcinoma	13	11.02±2.65	104.65±28.06	3.83±0.33	6.42±1.74
t	-	0.197	0.078	0.887	0.258
P	-	0.844	0.938	0.380	0.798
Depth of myometrial infiltration					
<1/2	35	9.91±2.93	95.41±25.58	3.73±0.38	5.84±1.91
1/2	14	14.29±2.28	129.68±20.31	4.36±0.31	8.26±1.59
t	-	-5.008	-4.471	-5.504	-4.188
P	-	<0.001	<0.001	<0.001	<0.001

### The correlation between serum Th1/Th2 cytokine levels and tumour marker levels in patients with HR-HPV-positive cervical cancer

According to the findings of the Pearson correlation analysis, there was a positive association (P<0.05) between the levels of SCC-Ag, CA199, and CYFRA21-1 and the blood levels of IL-10, IL-6, IFN-γ, and TNF-α in patients with HR-HPV-positive cervical cancer. See Table 6.

**Table 6:** Correlation between serum Th1/Th2 cytokine levels and tumour marker levels in patients with HR-HPV-positive cervical cancer.

Indicator	SCC-Ag		CA199		CYFRA21-1	
	r	p	r	p	r	p
IL-10	0.391	0.004	0.389	0.007	0.473	<0.001
IL-6	0.465	<0.001	0.422	<0.001	0.541	<0.001
IFN-γ	0.472	<0.001	0.458	<0.001	0.559	<0.001
TNF-α	0.497	<0.001	0.534	<0.001	0.576	<0.001

### Value of serum Th1/Th2 cytokines and tumour markers in the diagnosis of HR-HPV-positive cervical cancer

The occurrence of HR-HPV-positive cervical cancer was used as the status variable (no=0, yes=1). ROC curves were plotted. The AUCs of IL-10, IL-6, IFN-γ, and TNF-α for the single diagnosis of HR-HPV-positive cervical cancer were 0.809, 0.773, 0.801, and 0.794, respectively. The AUCs of SCC-Ag, cA199 and CYFRA21-1 for the single diagnosis of HR-HPV-positive cervical cancer were 0.831, 0.728 and 0.789, respectively.

The AUC of the combined diagnosis of HR-HPV-positive cervical cancer by serum IL-10, IL-6, IFN-γ, TNF-α, SCC-Ag, CA199 and CYFRA21-1 was 0.927. The AUC of the combined diagnosis of HR-HPV-positive cervical cancer was 0.927, and the AUC of the combined diagnosis was greater than that of the individual diagnosis of each index (Z=2.116, 2.690, 2.341, 2.565, 1.957, 3.351, 2.631; P=0.034, 0.007, 0.019, 0.010, 0). 039, 0.001, 0.009). See Table 7.

**Table 7:** Value of serum Th1/Th2 cytokines and tumour markers in the diagnosis of HR-HPV positive cervical cancer.

Indicator	AUC	AUC<br>(95% CI)	Optimal<br>truncation value	Sensitivity<br>(%)	Specificity<br>(%)	Yoden Index	P
IL-10	0.809	0.717 0.882	10.90 pg/mL	73.47	83.67	0.571	<0.001
IL-6	0.773	0.677 0.851	92.30 pg/mL	71.43	73.47	0.449	<0.001
IFN-γ	0.801	0.709 0.875	3.70 pg/mL	73.45	89.80	0.633	<0.001
TNF-α	0.794	0.700 0.869	5.21 pg/mL	81.63	69.39	0.510	<0.001
SCC-Ag	0.831	0.742 0.899	3.03 ng/mL	63.27	93.88	0.572	<0.001
CA199	0.728	0.628 0.813	30.12 U/m L	67.35	75.51	0.429	<0.001
CYFRA21-1	0.789	0.695 0.865	6.10 ng/mL	79.59	67.35	0.469	<0.001
Seven <br>combinations	0.927	0.857 0.970	-	83.67	89.80	0.735	<0.001

## Discussion

Serological markers have the advantages of convenient detection, stable results, economy and practicality and are currently a research hotspot in tumour diagnosis [9][10][11]. Serum SCC-Ag, CA199 and CYFRA21-1 are commonly used tumour markers for the clinical diagnosis of cervical cancer [12][13][14]. This study also revealed that, compared with those in HR-HPV-positive noncervical cancer patients, the levels of serum IL-10, IL-6, IFN-γ, and TNF-α in HR-HPV-positive cervical cancer patients were significantly increased, indicating that the elevated serum IL-10, IL-6, IFN-γ, and TNF-α are also related to the occurrence of HR-HPV-positive cervical cancer [15][16]. This difference lies in the fact that the Th1/Th2 balance plays a vital role in maintaining the immune status of the body [17][18]. Positive HR-HPV leads to an imbalance in Th1/Th2, with the Th1/Th2 balance shifting toward Th2. The immunosuppressive effect of Th2 is greater than that of Th1, resulting in a decrease in the body's antitumour effect. Eventually, it promotes the growth of cancer cells and leads to the occurrence of cervical cancer [19]. Relevant studies [20][21] have shown that elevated levels of serum IL-10, IL-6, IFN-γ, and TNF-α are significantly associated with the occurrence of HR-HPV-positive cervical cancer, which is consistent with the results of this study. Further analysis revealed that there were significant differences in the levels of serum IL-10, IL-6, IFN-γ, and TNF-α among HR-HPV-positive cervical cancer patients with different FIGO stages and invasion depths. These findings indicate that the shift in the Th1/Th2 balance to Th2 is not only an essential link in the occurrence of HR-HPV-positive cervical cancer but also related to the progression of cervical cancer.

The AUCs of serum IL-10, IL-6, IFN-γ, and TNF-α for the diagnosis of HR-HPV-positive cervical cancer are 0.809, 0.773, 0.801, and 0.794, respectively, which are all above 0.7 and have value for the diagnosis of HR-HPV-positive cervical cancer. However, the diagnostic value of each indicator applied alone is not high, and each indicator needs to be combined with other indicators. This study revealed that the AUC of serum IL-10, IL-6, IFN-γ, and TNF-α combined with SCC-Ag, CA199, and CYFRA21-1 for the diagnosis of HR-HPV-positive cervical cancer increased to 0.927, which was significantly greater than the AUC of each index diagnosed alone and could provide a more accurate reference basis for the clinical diagnosis of HR-HPV-positive cervical cancer. The reason might be that the combination of serum Th1/Th2 cytokines and tumour markers can reflect the occurrence of cervical cancer in terms of immune balance and specific changes in tumour cells, providing more comprehensive information for the diagnosis of HR-HPV-positive cervical cancer and helping to improve diagnostic efficiency.

The results of this study revealed statistically significant differences in the age of menarche ≤13 years and the imbalance of vaginal microecology between the study group and the control group (P<0.05). Furthermore, previous research has shown that a menarche age of >13 years and a vaginal microecological imbalance are also independent risk factors for the occurrence of HR-HPV-positive cervical cancer [22][23]. Therefore, for HR-HPV-positive patients with a menarche age of >13 years and vaginal microecological imbalance, cervical cancer prevention and intervention should be strengthened. However, the diagnostic value of a male age >13 years and a vaginal microecological imbalance for HR-HPV-positive cervical cancer patients has not been clearly defined [24][25][26]. This is also a deficiency of this study, and further in-depth exploration is still needed in future work.

In summary, Serum IL-10, IL-6, IFN-γ, and TNF-α levels were markedly elevated in individuals with HR-HPV-positive cervical cancer. Each of these variables had a specific level of diagnostic effectiveness in identifying HR-HPV-positive cervical cancer and was an independent risk factor for its development.

## Dodatak

### Availability of data and materials

The datasets generated or analysed during the current study are not publicly available because they contain private information. However, the data are available from the corresponding author upon reasonable request.

Xia Cao and Xuemei Zhang have made the same contribution to this work.

### Conflict of interest statement

All the authors declare that they have no conflict of interest in this work.
